# Circ‐AKT3 inhibits the accumulation of extracellular matrix of mesangial cells in diabetic nephropathy via modulating miR‐296‐3p/E‐cadherin signals

**DOI:** 10.1111/jcmm.15513

**Published:** 2020-06-28

**Authors:** Bo Tang, Weiliang Li, Ting‐Ting Ji, Xiao‐Ying Li, Xiaolei Qu, Linhong Feng, Shoujun Bai

**Affiliations:** ^1^ Department of Nephrology Qingpu Branch of Zhongshan Hospital Affiliated to Fudan University Shanghai China

**Keywords:** circ‐AKT3, diabetic nephropathy, E‐cadherin, miR‐296‐3p

## Abstract

Diabetic nephropathy is a leading cause of end‐stage renal disease globally. The vital role of circular RNAs (circRNAs) has been reported in diabetic nephropathy progression, but the molecular mechanism linking diabetic nephropathy to circRNAs remains elusive. In this study, we investigated the significant function of circ‐AKT3/miR‐296‐3p/E‐cadherin regulatory network on the extracellular matrix accumulation in mesangial cells in diabetic nephropathy. The expression of circ‐AKT3 and fibrosis‐associated proteins, including fibronectin, collagen type I and collagen type IV, was assessed via RT‐PCR and Western blot analysis in diabetic nephropathy animal model and mouse mesangial SV40‐MES13 cells. Luciferase reporter assays were used to investigate interactions among E‐cadherin, circ‐AKT3 and miR‐296‐3p in mouse mesangial SV40‐MES13 cells. Cell apoptosis was evaluated via flow cytometry. The level of circ‐AKT3 was significantly lower in diabetic nephropathy mice model group and mouse mesangial SV40‐MES13 cells treated with high‐concentration (25 mmol/L) glucose. In addition, circ‐AKT3 overexpression inhibited the level of fibrosis‐associated protein, such as fibronectin, collagen type I and collagen type IV. Circ‐AKT3 overexpression also inhibited the apoptosis of mouse mesangial SV40‐MES13 cells treated with high glucose. Luciferase reporter assay and bioinformatics tools identified that circ‐AKT3 could act as a sponge of miR‐296‐3p and E‐cadherin was the miR‐296‐3p direct target. Moreover, circ‐AKT3/miR‐296‐3p/E‐cadherin modulated the extracellular matrix of mouse mesangial cells in high‐concentration (25 mmol/L) glucose, inhibiting the synthesis of related extracellular matrix protein. In conclusion, circ‐AKT3 inhibited the extracellular matrix accumulation in diabetic nephropathy mesangial cells through modulating miR‐296‐3p/E‐cadherin signals, which might offer novel potential opportunities for clinical diagnosis targets and therapeutic biomarkers for diabetic nephropathy.

## 1 INTRODUCTION

Diabetic nephropathy is a lethal diabetic complication and the primary (accounting for 30%‐47%) cause of end‐stage renal disease around the world.^1^, ^2^ The pathogenesis of diabetic nephropathy is characterized by a complicated interplay between hemodynamic and metabolic disruptions.^3^ The metabolic damage caused by hyperglycaemia is vital to the diabetic nephropathy development.^1^ The pathological features of diabetic nephropathy are accumulated extracellular matrix and proteins, including fibronectin (FN) and collagen, thickened glomerular membranes, as well as progressive mesangial hypertrophy.^4^ For diabetic patients, mesangial cells exposed to high‐concentration glucose conditions lead to the ectopic expression of fibrins and cytokine, which results in renal fibrosis in return.^5^ Therefore, it is crucial to examine the potential underlying molecular mechanism of high glucose in the progression of diabetic nephropathy.

Circular RNAs (circRNAs) belong to a novel type of non‐coding RNA, which is characterized by the presence of covalent bonds that connect the 3 'and 5' ends via back splicing.^6^, ^7^ CircRNAs function as cytoplasmic RNA‐binding protein chelators and microRNA (miRNA) sponges, and the regulators of nuclear transcription, indicating the significance of circRNAs as participants in the governing of gene expression.^8^ Recently, circRNAs have been investigated in the development of numerous diseases, including cancer, diabetes, neurological disorders and heart disease.^9^ Current studies have suggested the vital effect of the ‘circRNA/miRNA/mRNA’ on multiple development pathways and disease pathogenesis like diabetic nephropathy.^10^ For instance, the study conducted by Liu et al^11^ indicated that cell proliferation and fibrosis were suppressed by circ_0080425 through the sponge of miR‐24‐3p and the target of fibroblast growth factor 11 in diabetic nephropathy. Additionally, the study of Xue et al^12^ found that the metastasis of clear cell renal cell carcinoma was inhibited by circ‐AKT3 through the regulation of E‐cadherin and miR‐296‐3p signalling. However, the circ‐AKT3 role of diabetic nephropathy progression is still not well‐documented.

MiRNAs, containing 20‐22 nucleotides, are small non‐coding RNAs capable of target mRNA degradation by binding to their 3' untranslated regions (UTR).^13^ Various miRNAs (such as miR21, miR192 and miR‐1207‐5p) involved in diabetic nephropathy have been confirmed.^14^ Previous studies found that miR21, miR192 and miR‐1207‐5p were up‐regulated in TGF‐β1‐treated mouse mesangial cells and glomeruli in diabetic mouse models as compared to non‐diabetic control mice,^14^, ^15^, ^16^ which may be considered as diagnostic or therapeutic biomarkers.^17^ However, the roles of miRNAs in the diabetic nephropathy development are still not well discovered.

To date, the interaction of circRNA‐miRNA‐mRNA on diabetic nephropathy progression remains unclear. In this study, we investigate the significant circ‐AKT3/miR‐296‐3p/E‐cadherin axis function on the extracellular matrix of mesangial cells in diabetic nephropathy progression, which provides important insights into the management of the disease, especially in early diagnosis and treatment.

## 2 METHODS

### 2.1 Diabetic nephropathy animal models

We purchased spontaneous diabetic db/db mice (C57BL/KsJ‐db/db) and corresponding non‐diabetic db/m mice from Shanghai SLAC Laboratory Animal Co., Ltd. All animals were caged in a standard temperature‐controlled laboratory in a 12/12 h light/dark cycle, with unlimited access to water and food. This study was conducted in conformity to protocols approved by the Ethics Committee of the Animal Research Institute of Qingpu Branch of Zhongshan Hospital Affiliated to Fudan University. The glomerular fraction of the kidneys extracted from mice was used in this study.

### 2.2 Cell culture

Mouse mesangial cells (SV40‐MES13) were obtained from the Shanghai Academy of Life Sciences. They were grown in Dulbecco's Modified Eagle's Medium (DMEM; Invitrogen) supplemented with glutamine (2 μmmol/L), 20% foetal calf serum (FBS; Gibco), β‐mercaptoethanol (50 μmmol/L) and 2.5% streptomycin and penicillin under a humidified atmosphere of 5% CO_2_ at 37°C. The growth of diabetic nephropathy cells was simulated with high‐glucose treatment. In short, mouse mesangial cells were cultured with normal medium (5.5 mmol/L d‐glucose) or high glucose (25 mmol/L d‐glucose).

### 2.3 Lentiviral transduction

The sequences of miR‐296‐3p mimic and circ‐AKT3 obtained from RiboBio were presented in Table S1. The lentivirus overexpression circ‐AKT3 plasmid was produced by GenePharm. The supernatant containing lentivirus particles was harvested after 48 hours and was employed to transduce mouse mesangial cells. After 24 hours, 2 μg/mL puromycin was added to select stably transduced cells.

### 2.4 Total RNA preparation, reverse transcription and RT‐PCR

Total RNA was obtained from cultured cells with the use of RNAiso Plus as the manufacturer's protocols. Next, we used PrimeScript™ RT Master Mix to reversely transcribe 1 µg of RNA to complementary DNA. Finally, the RT‐PCR was conducted with the use of TB Green® Premix Ex Taq™ based on the standard protocol, with the aim of detecting circRNA and mRNA expression. The internal control for mRNA and circRNA mRNA was GAPDH and U6, respectively. The 2^−ΔΔCt^ method was performed to calculate the relative expression. All the specific primers were presented in Table 1.

**Table 1:** The primers used in this study

Primers	
circ‐AKT3 (Forward primer)	TCCTTCCAGACAAAAGACCGT
circ‐AKT3 (Reverse primer)	CGCTCATGATGACTCCCCTC
GAPDH (Forward primer)	GCACAAACGAGGGGAGTACA
GAPDH (Reverse primer)	AAATGAGCCCCAGCCTTCTC
E‐cadherin (Forward primer)	GTCTGTAGGAAGGCACAGCC
E‐cadherin (Reverse primer)	TCATCCTCTGGGGGCAGTAA
miR‐296‐3p	GAGGGTTGGGTGGAGGCTCTCC
U6 (Forward primer)	GAAGTTGTTCGTGGTGGATTCG
U6 (Reverse primer)	CCTCTGGGCCCTTCCTCCAG

### 2.5 Western blot analysis

Proteins were extracted according to the previous method and separated on 8%‐10% SDS/PAGE gel.^18^ Then, proteins were transferred to PVDF membranes (Bio‐Rad). Non‐fat milk was used to block membranes, which were incubated with antibodies. Anti‐E‐cadherin (1:1000), anti‐collagen type I (Col. I) (1:1000), anti‐Col. IV (1:1000), anti‐FN (1:1000), anti‐Cleaved caspase 3 (1:1000), anti‐Caspase 3 (1:1000), anti‐Bax (1:1000), anti‐Bcl‐2 (1:1000) and GAPDH (1:1000) antibodies were obtained from Abcam. After the membranes were incubated overnight at 4°C, they were incubated with secondary antibodies for 1 hour at room temperature. The ECL kit purchased from Amersham Biosciences was utilized to visualize the immunocomplexes.

### 2.6 Luciferase report assay

We used a Dual‐Luciferase Reporter kit (Promega) to conduct the luciferase assay. The pGL3 vector (Promega) was utilized to clone the wild‐type (WT) or mutant type (MUT) of 3’‐UTR of E‐cadherin and circ‐AKT3. In addition, mouse mesangial cells were cotransfected with miR‐296‐3p mimics or negative control by Lipofectamine 2000 (Invitrogen) based on the manufacturers’ instructions. The luminescence was determined using a Microplate Reader (PerkinElmer), after 48‐hour transfection.

### 2.7 Histological analysis

The kidney tissues of mice in each group were fixed with 10% paraformaldehyde for 48 hours, then embedded in paraffin and cut into 4 μm sections. Next, kidney tissue was stained with haematoxylin‐eosin (HE), and the morphological changes in kidney tissue were examined under an inverted phase‐contrast microscope. The interstitial injury score was assessed via researchers who were not familiar with the present study.

### 2.8 Flow cytometry

Cell apoptosis was assessed utilizing the Annexin V‐FITC Apoptosis Detection Kit (Abcam) following the manufacturer's instructions. Briefly, cells in each transfection group were suspended in 500 μL of binding buffer, and then, 5 μL of Annexin V‐/FITC and PI was added. Cells were fostered at room temperature for 15 minutes in the dark. Apoptosis was evaluated via BD FACS software.

### 2.9 Statistical analysis

The data in this study were displayed as mean ± SEM. SPSS 25.0 was used to perform statistical analysis, using one‐way ANOVA followed by Bonferroni's multiple comparisons or Student's *t* test for the group comparisons. Statistically significant difference was considered when *P* < .05.

## 3 RESULTS

### 3.1 Circ‐AKT3 might be involved in diabetic nephropathy progression

**Figure 1:**
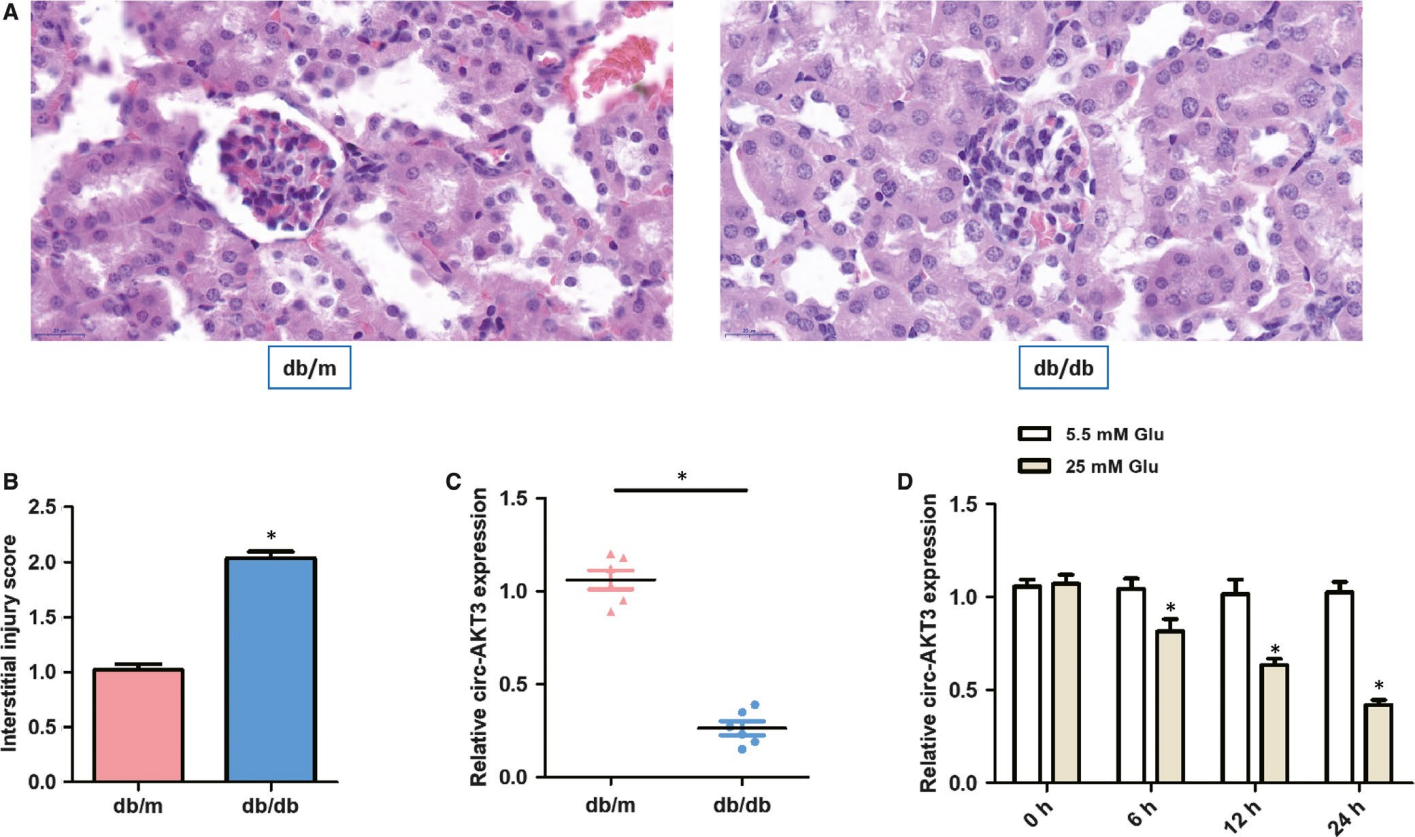
Circ‐AKT3 might be involved in diabetic nephropathy progression. (A) Representative histological images in two groups. (B) Interstitial injury score in two groups. (C) The relative circ‐AKT3 expression was measured by RT‐PCR in diabetic nephropathy db/db mice compared with matched normal db/m mice (n = 6). (D) The relative expression of circ‐AKT3 was determined by RT‐PCR in mouse mesangial cells (SV40‐MES13) exposed to normal glucose (5.5 mmol/L Glu) and high glucose (25 mmol/L Glu). *P < .05

To examine histopathological changes of glomerular fraction of the kidneys extracted from mice, HE staining was utilized in the present study. Figure 1A showed morphologically normal glomerular tissue in the normal db/m mice group, which a diffuse thickening of the glomerular basement membrane and an increase in the mesangial matrix occurred in db/db mice group. As presented in Figure 1B, the db/db mice group reported significantly higher interstitial injury scores than db/m mice group. These data manifested that the diabetic nephropathy mice model was successfully established in this study.

In addition, to investigate the potential circ‐AKT3 role on diabetic nephropathy progression, RT‐PCR was performed to compare the relative circ‐AKT3 expression in diabetic nephropathy db/db mice with that of the compared normal db/m mice (n = 6). As presented in Figure 1C, the circ‐AKT3 expression was remarkably decreased in db/db mice group, compared to the matched normal db/m mice group.

We exposed mouse mesangial SV40‐MES13 cells to high‐concentration (25 mmol/L) glucose and normal (5.5 mmol/L Glu) glucose in vitro. The RT‐PCR results revealed that the relative circ‐AKT3 level was remarkably down‐regulated in the 25 mmol/L Glu group at different treatment times, which showed a time‐dependence characteristic, as displayed in Figure 1D. Therefore, these data revealed that the lower circ‐AKT3 expression might be involved in diabetic nephropathy progression.

### 3.2 The overexpression of circ‐AKT3 inhibited the exposed with normal extracellular matrix accumulation of mouse mesangial cells

**Figure 2:**
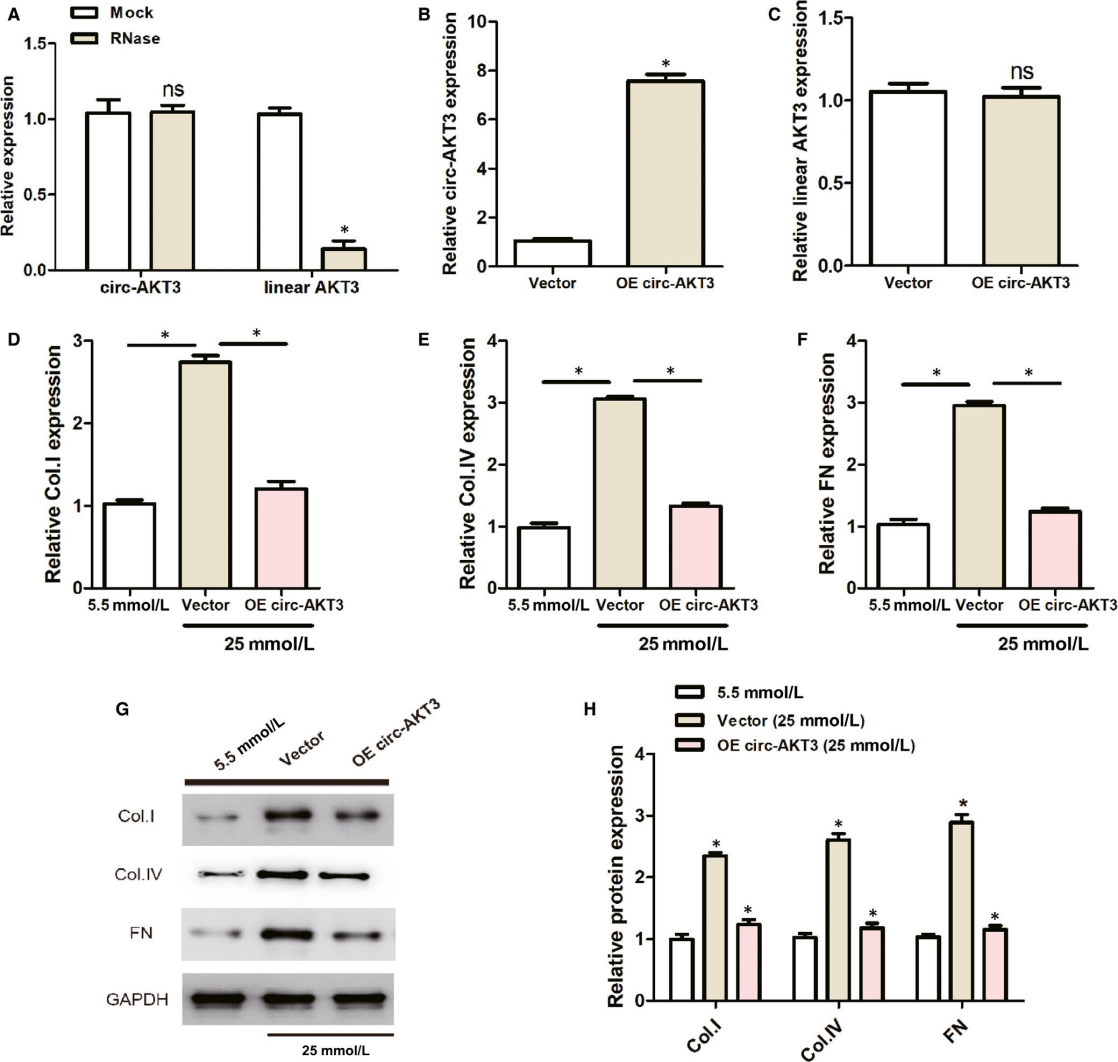
The overexpression of circ‐AKT3 suppressed the exposed with normal extracellular matrix accumulation of mouse mesangial cells. (A‐C) The overexpression circ‐AKT3 lentivirus plasmid was constructed and its transfection efficiency was confirmed by RT‐PCR. (D‐F) RT‐PCR results indicated the expression levels of Col. I, Col. IV and FN in mouse mesangial cells treated with normal glucose (5.5 mmol/L) or high glucose (25 mmol/L) or transfected with OE circ‐AKT3. (G, H) Relative protein expression of Col. I, Col. IV and FN in mouse mesangial cells treated with normal glucose (5.5 mmol/L) or high glucose (25 mmol/L) or transfected with OE circ‐AKT3. *P < .05. collagen type I (Col. I), collagen type IV (Col. IV) and fibronectin (FN)

As demonstrated in Figure 2A, RT‐PCR results testified that no significant difference was found in the expression of circ‐AKT3 between the Mock group and RNase group. However, compared with the Mock group, the RNase group had a significantly higher level of linear AKT3. As presented in Figure 2B, the relative circ‐AKT3 expression was remarkably higher in the OE circ‐AKT3 group than that of the Vector group, which indicated the efficient transfection of OE circ‐AKT3 lentivirus plasmid. Figure 2C suggested that no significant difference was observed in relative linear AKT3 expression between the Vector group and the OE circ‐AKT3 group. These results manifested that only circ‐AKT3 was successfully overexpressed and silenced in cells.

RT‐PCR and Western blot analysis were conducted to determine the relative mRNA and protein expression of fibrosis‐associated proteins, such as FN, Col. I and Col. IV in the mouse mesangial SV40‐MES13cells treated with high glucose. As can be seen from Figure 2D‐H, the mRNA and protein expressions of FN, Col. I and Col. IV were remarkably increased in the Vector group treated with high‐concentration (25 mmol/L) glucose, compared to the normal (5.5 mmol/L Glu) glucose group. However, there was a significant decrease in the mRNA and protein expression levels of FN, Col. I and Col. IV in the OE circ‐AKT3 transfection group, compared to the Vector group treated with high glucose. Thus, the overexpressed circ‐AKT3 inhibited the mRNA and protein level of fibrosis‐related protein, indicating the effect of circ‐AKT3 on extracellular matrix accumulation.

### 3.3 Circ‐AKT3 overexpression inhibited the apoptosis of mouse mesangial SV40‐MES13 cells treated with high glucose

**Figure 3:**
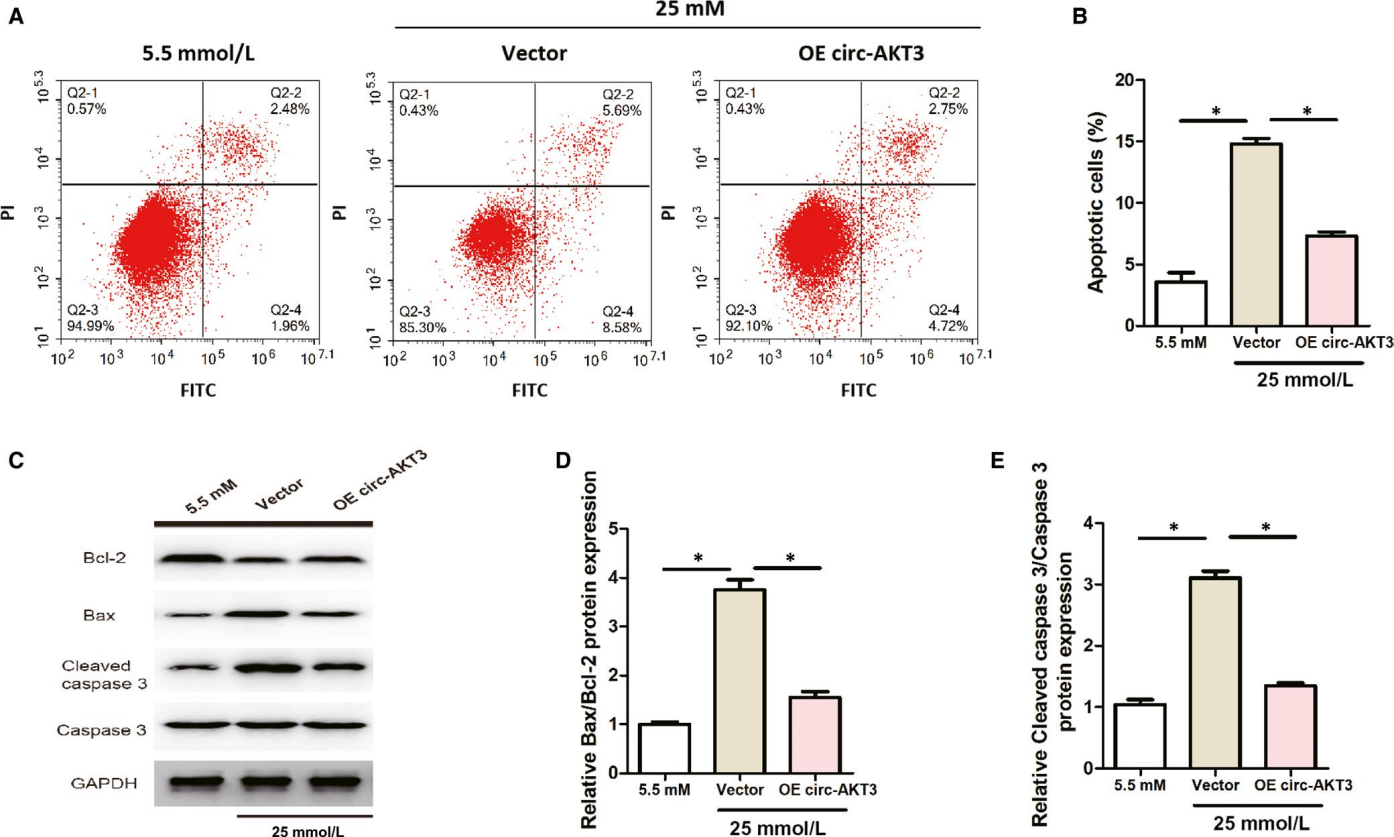
Circ‐AKT3 overexpression inhibited the apoptosis of mouse mesangial SV40‐MES13 cells treated with high glucose. (A, B) Cell apoptosis of mouse mesangial SV40‐MES13 cells was detected by flow cytometry assay. (C) Bax, Bcl‐2, Caspase 3 and Cleaved caspase 3 protein expression were measured by Western blot analysis in each group. (D, E) Relative Bax/Bcl‐2 and Cleaved caspase 3/Caspase 3 protein expression were assessed by RT‐PCR in each group. *P < .05

As shown in Figure 3A,B, cell apoptosis was significantly inhibited after overexpressing circ‐AKT3 in high‐glucose–treated mouse mesangial SV40‐MES13 cells. Figure 3C‐E indicated that overexpressing circ‐AKT3 significantly reduced relative Cleaved caspase 3/Caspase 3 protein expression and relative Bax/Bcl‐2 protein expression in mouse mesangial SV40‐MES13 cells exposed to high glucose. These results suggested that circ‐AKT3 overexpression inhibited the apoptosis of mouse mesangial SV40‐MES13 cells treated with high glucose.

### 3.4 miR‐296‐3p was confirmed to target circ‐AKT3 and E‐cadherin acted as the target protein of miR‐296‐3p

**Figure 4:**
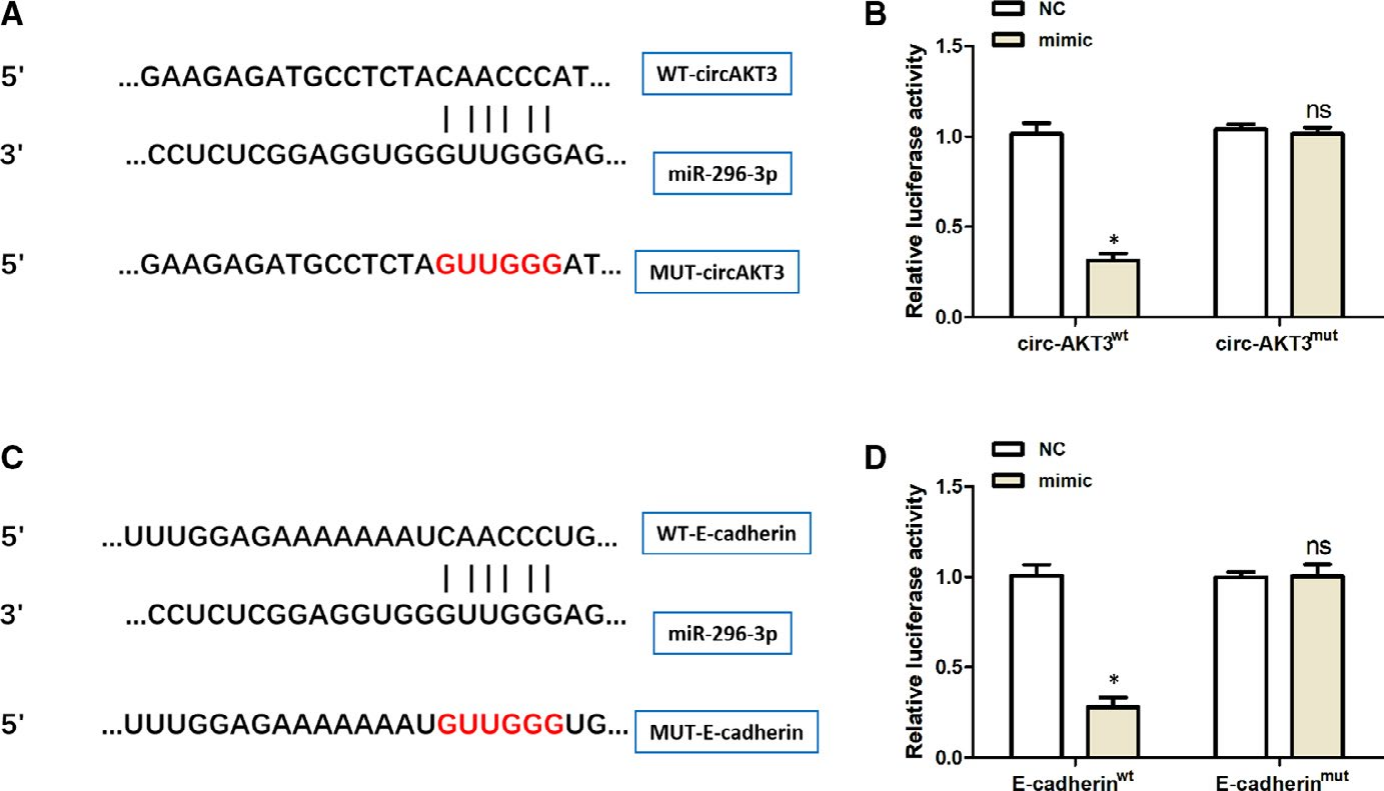
miR‐296‐3p was confirmed to target circ‐AKT3 and E‐cadherin acted as the target protein of miR‐296‐3p. (A) Schematic representation of binding sites between miR‐296‐3p and circ‐AKT3. (B) Relative luciferase activities in mouse mesangial SV40‐MES13 cells cotransfected with the miR‐296‐3p mimic or negative control containing either circ‐AKT3 wild‐type or circ‐AKT3 mutant type. (C) Schematic representation of binding sites between miR‐296‐3p and E‐cadherin mRNA. (D) Relative luciferase activities in mouse mesangial SV40‐MES13 cells cotransfected with the miR‐296‐3p mimic or negative control containing either E‐cadherin mRNA wild‐type or E‐cadherin mRNA mutant type. *P < .05

**Figure 5:**
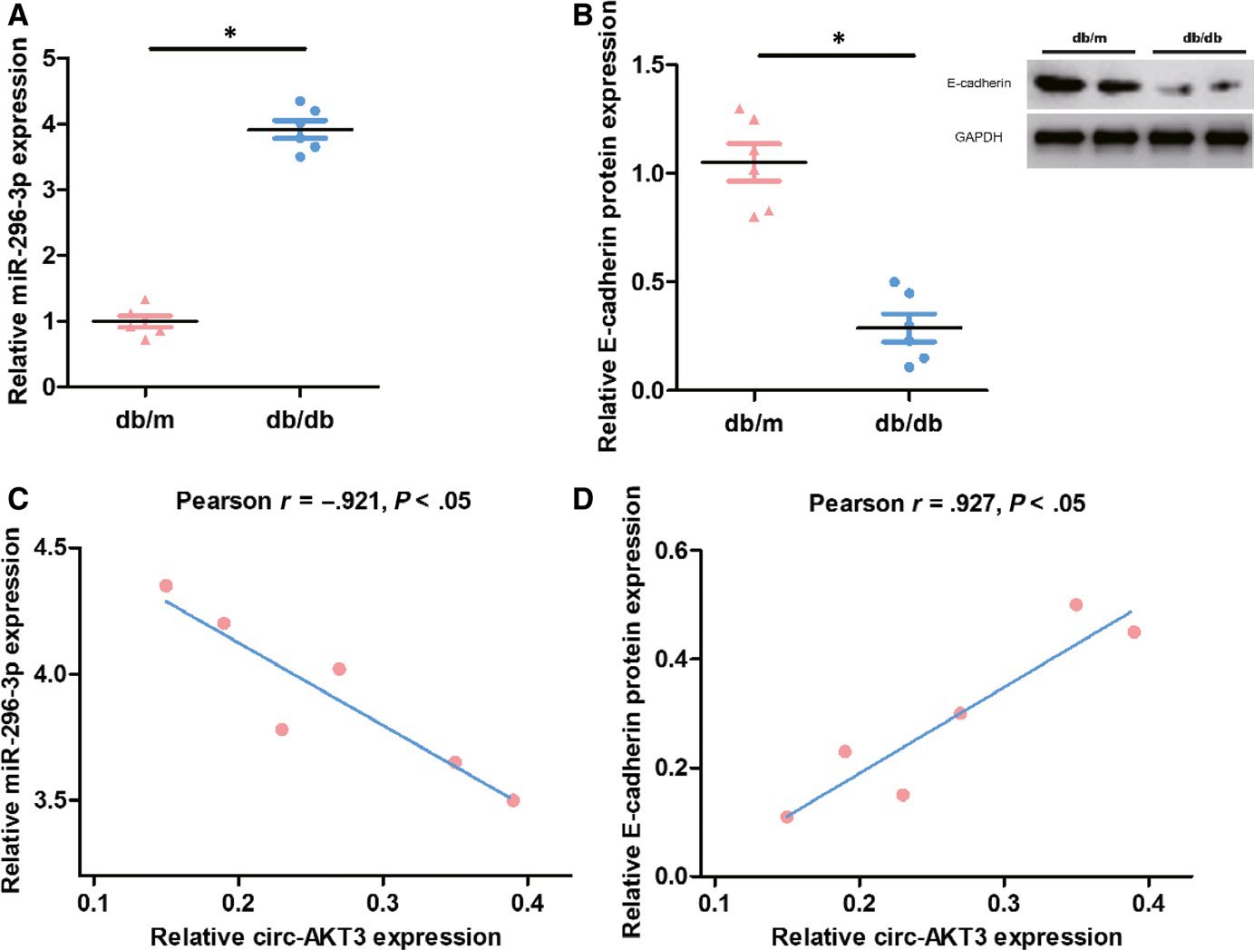
The correlation between circ‐AKT3, miR‐296‐3p and E‐cadherin. (A) The relative miR‐296‐3p expression was assessed by RT‐PCR in diabetic nephropathy db/db mice compared with matched normal db/m mice (n = 6). (B) The relative E‐cadherin mRNA and protein expression were evaluated by RT‐PCR and Western blot analysis in diabetic nephropathy db/db mice compared with matched normal db/m mice (n = 6). (C) Pearson's correlation analysis of circ‐AKT3 and miR‐296‐3p expressions in db/db diabetic nephropathy mice. (D) Pearson's correlation analysis of circ‐AKT3 and E‐cadherin expressions in db/db diabetic nephropathy mice.*P < .05

To explore the potential interaction between miR‐296‐3p and circ‐AKT3, we inserted the WT and MUT predicted binding site sequences of circ‐AKT3 into the luciferase reporter vector pmirGLO in Figure 4A. The relative luciferase activity was dramatically decreased in mouse mesangial cells (SV40‐MES13) transfected with the sequence of circ‐AKT3^wt^, whereas the luciferase activity did not show a significant difference in the circ‐AKT3^mut^ group, compared to the negative control (NC) group, as presented in Figure 4B. RT‐PCR results from Figure 5A revealed that miR‐296‐3p expression was significantly higher in db/db diabetic nephropathy mice group as compared to the matched normal db/m mice group. As can be seen from Figure 5C, circ‐AKT3 was negatively correlated to miR‐296‐3p expression in db/db diabetic nephropathy mice by Pearson's correlation analysis. These results revealed that circ‐AKT3, through the expected binding site, acted as a sponge of miR‐296‐3p.

Further, we constructed the luciferase reporter plasmids containing the 3’UTR of E‐cadherin^WT^ or E‐cadherin^mut^ into in mouse mesangial cells (SV40‐MES13), so as to explore the probable function between miR‐296‐3p and E‐cadherin mRNA, as displayed in Figure 4C. MiR‐296‐3p mimics could remarkably decrease the relative luciferase activity of those transfected with the reporter plasmid containing the 3’UTR of E‐cadherin^WT^, while no significant change was detected in the E‐cadherin^mut^ group, compared to the NC control, as can be seen from Figure 4D. RT‐PCR and Western blot analysis results from Figure 5B showed that E‐cadherin protein expression was significantly lower in db/db diabetic nephropathy mice group than that in the matched normal db/m mice group. As presented in Figure 5D, circ‐AKT3 was positively correlated to E‐cadherin expression in db/db diabetic nephropathy mice through Pearson's correlation analysis. These data suggested that miR‐296‐3p had the predicted binding site, which could directly bind to the 3’‐UTR of E‐cadherin mRNA. Taken together, we found that miR‐296‐3p was confirmed to target circ‐AKT3 and E‐cadherin acted as the target protein of miR‐296‐3p.

### 3.5 Regulation of circ‐AKT3/miR‐296‐3p/E‐cadherin network on the extracellular matrix of mouse mesangial cells

**Figure 6:**
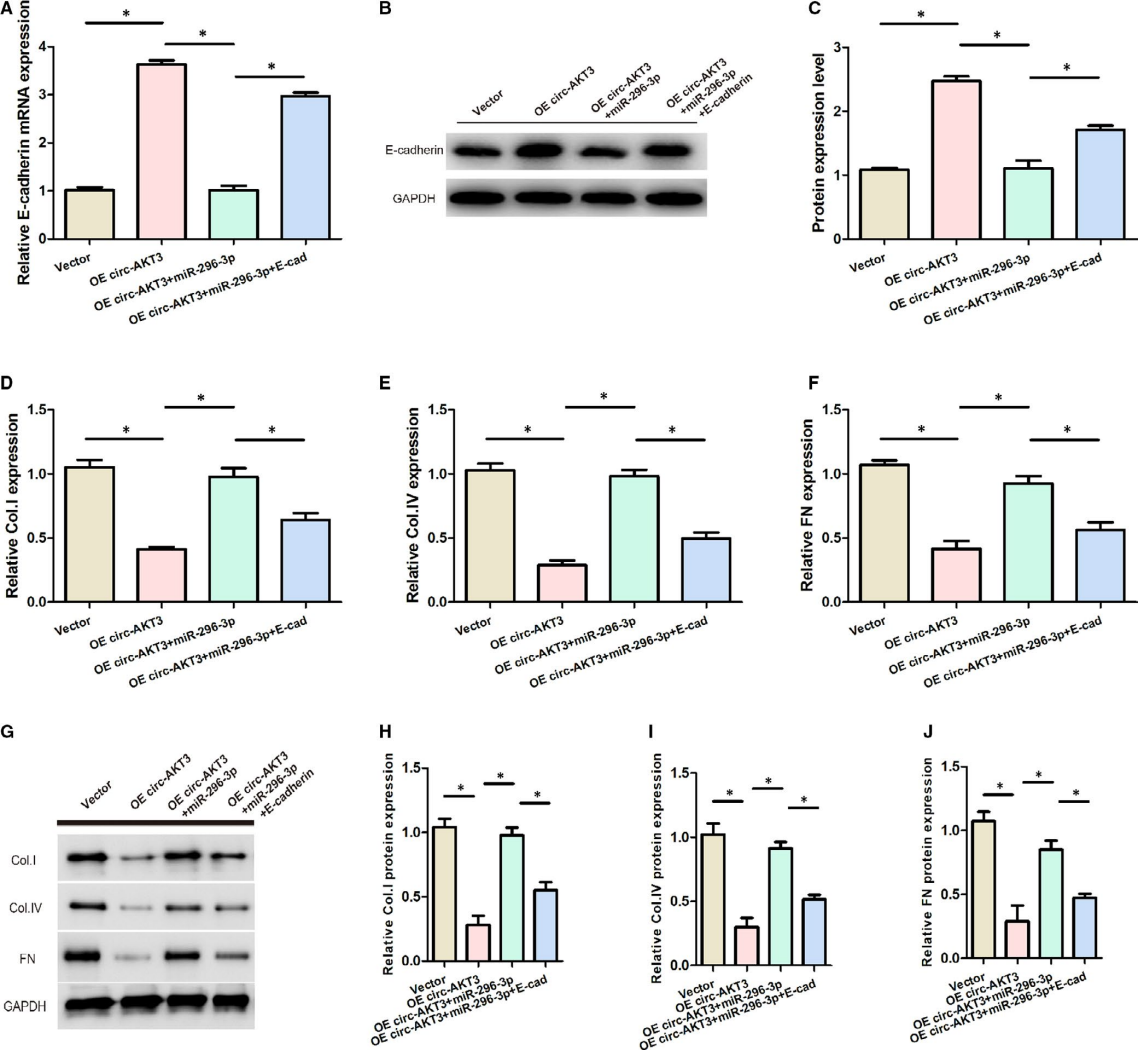
circ‐AKT3/miR‐296‐3p/E‐cadherin regulated the extracellular matrix of mouse mesangial cells in high glucose. (A) Relative expression of E‐cadherin mRNA in each group. (B, C) The expression of E‐cadherin protein in each group. (D) Relative Col. I expression in each group. (E) Relative Col. IV expression in each group. (F) Relative FN expression in each group. (G‐J) Relative protein expression of Col. I, Col. IV and FN in each group.*P < .05. collagen type I (Col. I), collagen type IV (Col. IV) and fibronectin (FN)

The experimental data from RT‐PCR and Western blot analysis indicated that the overexpression of circ‐AKT3 markedly up‐regulated E‐cadherin mRNA and protein levels, as displayed in Figure 6A‐C. However, the expression levels of E‐cadherin mRNA and protein were remarkably decreased in the OE circ‐AKT3 transfected with miR‐296‐3p group, and increased in the OE circ‐AKT3 transfected with E‐cadherin and miR‐296‐3p group, as presented in Figure 6A‐C. In addition, the relative mRNA and protein expressions of FN, Col. I and Col. IV were remarkably down‐regulated in the transfection of overexpressed circ‐AKT3 and up‐regulated in miR‐296‐3p transfection, as presented in Figure 6D‐J. As demonstrated in Figure 6D‐J, the relative mRNA and protein expressions of FN, Col. I and Col. IV were significantly lower in the OE circ‐AKT3 + miR‐296‐3p + E‐cadherin group in comparison with the OE circ‐AKT3 + miR‐296‐3p group. Therefore, these results showed that circ‐AKT3/miR‐296‐3p/E‐cadherin network modulated the extracellular matrix of mouse mesangial cells in high‐concentration glucose, inhibiting the synthesis of related extracellular matrix protein.

### 3.6 Effect of circ‐AKT3/miR‐296‐3p/E‐cadherin on the apoptosis of mouse mesangial SV40‐MES13 cells

**Figure 7:**
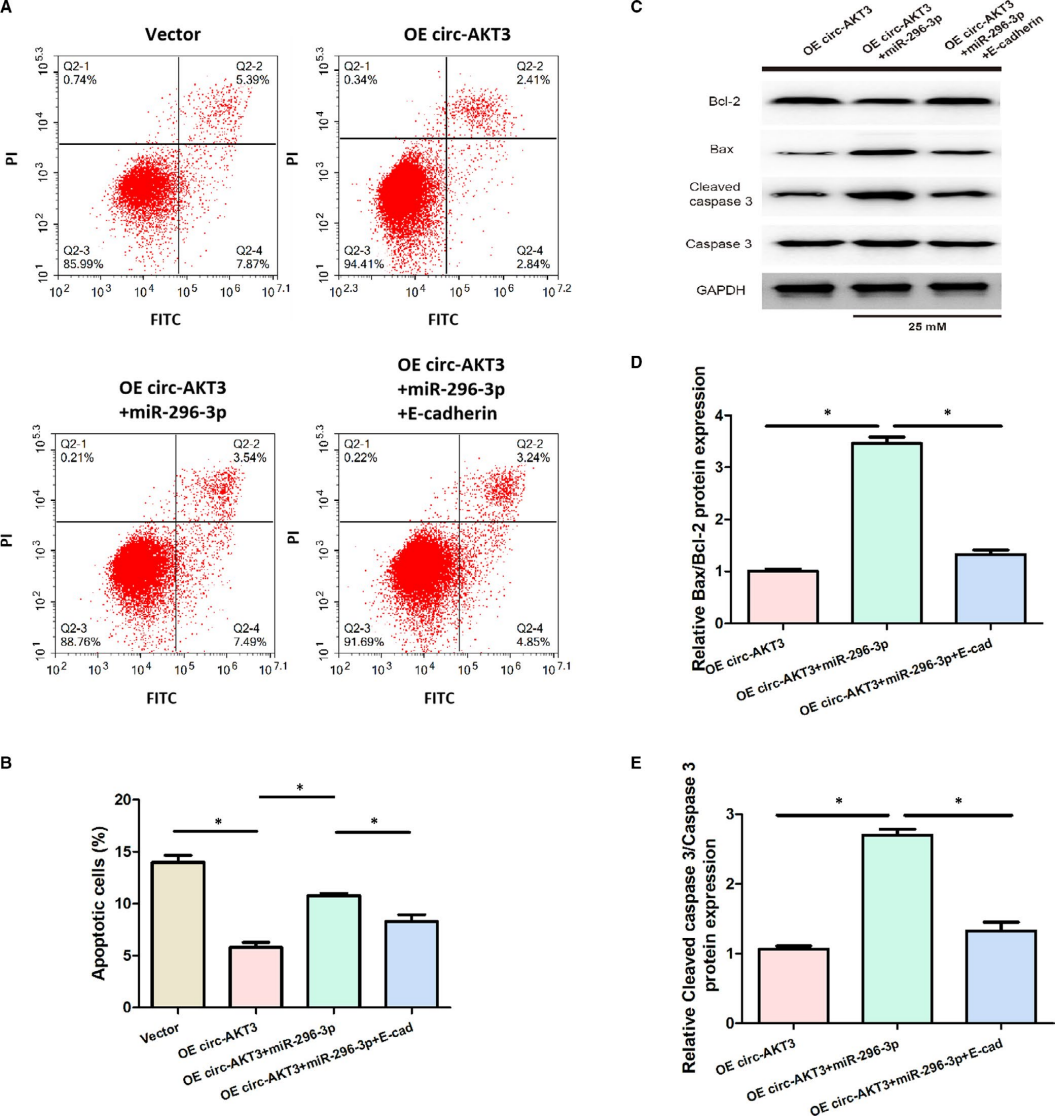
Effect of circ‐AKT3/miR‐296‐3p/E‐cadherin on the apoptosis of mouse mesangial SV40‐MES13 cells. (A, B) Cell apoptosis of mouse mesangial SV40‐MES13 cells was detected by flow cytometry assay in each group. (C‐E) Relative Bax/Bcl‐2 and Cleaved caspase 3/Caspase 3 protein expression were measured via Western blot analysis in each group *P < .05

As presented in Figure 7A,B, cell apoptosis was significantly reduced after overexpressing circ‐AKT3 in mouse mesangial SV40‐MES13 cells under high glucose condition, compared with the Vector group. Cell apoptosis was remarkably increased in the OE circ‐AKT3 transfected with miR‐296‐3p group, which was rescued by E‐cadherin.

Furthermore, Figure 7C‐E displayed that miR‐296‐3p significantly increased relative Bax/Bcl‐2 and Cleaved caspase 3/Caspase 3 protein expression induced by the overexpression of circ‐AKT3 in mouse mesangial SV40‐MES13 cells treated with high glucose, which was rescued by E‐cadherin. These results indicated that circ‐AKT3 suppressed the apoptosis of mouse mesangial SV40‐MES13 cells treated with high glucose via regulating miR‐296‐3p/E‐cadherin signals.

## 4 DISCUSSION

At present, the treatment of diabetic nephropathy is still a huge challenge, so the mechanism of diabetic nephropathy has received increasing attention.^19^ Cumulative evidence indicates circRNAs’ vital role in the progression of numerous diseases, including neurological diseases, diabetes and cancer.^20^In the present study, we expected to explore the circ‐AKT3 effect on a diabetic nephropathy mouse model and identify the regulation of circ‐AKT3/miR‐296‐3p/E‐cadherin network in the physiology and pathology of diabetic nephropathy.

In this study, we found that the relative circ‐AKT3 expression was remarkably down‐regulated in high‐concentration (25 mmol/L) glucose group. Also, the relative expression of circ‐AKT3 was decreased in diabetic nephropathy db/db mice model. Moreover, the overexpressed circ‐AKT3 inhibited the levels of fibrosis‐associated protein, such as FN, Col. I and Col. IV. Circ‐AKT3 overexpression also inhibited the apoptosis of mouse mesangial SV40‐MES13 cells treated with high glucose. Our study showed that miR‐296‐3p directly targeted circ‐AKT3 with the help of bioinformatics tools, and the luciferase report assay confirmed this finding. Here, we verified that circ‐AKT3 could act as a sponge of miR‐296‐3p for the first time.

In addition, this study indicated that E‐cadherin was the miR‐296‐3p direct target. Further, this association was verified by the luciferase report assay. E‐cadherin significantly reduced the synthesis of related extracellular matrix protein, such as FN, Col. I and Col. IV, and entirely rescued the miR‐296‐3p effect on the extracellular matrix accumulation in mesangial cells in diabetic nephropathy. Previous studies found that the low E‐cadherin expression, a transmembrane glycoprotein, was associated with tumour and metastasis, and E‐cadherin was reduced at the end‐stage of diabetic nephropathy, which may predict possible diabetic nephropathy evolution towards cancer.^21^ Our finding was supported by a recent study revealed that serum periostin and E‐cadherin might be regarded as dependable biomarkers in the pathogenesis of the early stage of diabetic nephropathy.^22^ Moreover, our data suggested that the E‐cadherin expression was remarkably stimulated via circ‐AKT3 while inhibited via miR‐296‐3p. The co‐transfection of OE circ‐AKT3and miR‐296‐3p eliminated the promotion of circ‐AKT3 overexpression on the E‐cadherin expression. Hence, the competitive binding between miR‐296‐3p and circ‐AKT3 significantly decreased the overexpression of E‐cadherin caused by OE circ‐AKT3, which resulted in increased the level of FN, Col. I and Col. IV during diabetic nephropathy progression.

To the best of our knowledge, this is the first study to report the effect of circ‐AKT3/miR‐296‐3p/E‐cadherin regulatory network on the extracellular matrix accumulation in mesangial cells in diabetic nephropathy. Our study found that circ‐AKT3 suppressed the extracellular matrix accumulation in diabetic nephropathy mesangial cells via regulating miR‐296‐3p/E‐cadherin signals, which may provide a possible biomarker for the diagnosis and treatment of diabetic nephropathy. Further studies are still needed to investigate more about the role and mechanism of other signals in the therapy of diabetic nephropathy in animals and humans.

In conclusion, our findings revealed that circ‐AKT3 inhibited the extracellular matrix accumulation in mesangial cells in diabetic nephropathy through modulating miR‐296‐3p/E‐cadherin signals, which might offer novel potential opportunities for clinical diagnosis targets and therapeutic biomarkers for diabetic nephropathy.

## CONFLICT OF INTERESTS

The authors confirm that there are no conflicts of interest.

## AUTHOR CONTRIBUTION

**Bo Tang:** Conceptualization (equal); Writing‐original draft (equal). **Weiliang Li:** Conceptualization (equal); Writing‐original draft (equal). **Ting‐Ting Ji:** Formal analysis (equal). **Xiao‐Ying Li:** Investigation (equal); Project administration (equal). **Xiaolei Qu:** Formal analysis (equal). **Linhong Feng:** Investigation (equal); Project administration (equal). **Shoujun Bai:** Supervision (equal); Writing‐review & editing (equal).

## Supporting information

Table S1

## Data Availability

Data were available on request from the authors.
