# Supplementary material for: Circ‐AKT3 inhibits the accumulation of extracellular matrix of mesangial cells in diabetic nephropathy via modulating miR‐296‐3p/E‐cadherin signals
Source: J Cell Mol Med. 2020 Jun 28;24(15):8779–88. doi: 10.1111/jcmm.15513 (PMC7412430; doi:10.1111/jcmm.15513)
Supplement: Supplementary file 1 — Table S1 [file JCMM-24-8779-s001.docx]

**Table S1. The sequences used in this study**

| **Sequences** | |
| --- | --- |
| circ-AKT3 | CGAGGCTGAGTCATCACTA |
| miR-296-3p sense | CGUUUCGUGUGCCGGACGUCUCU |
| miR-296-3p anti-sense | CUCCCAACCCACCUCCGAGAGG |
